# Current challenges in the management of patients with sickle cell disease – A report of the Italian experience

**DOI:** 10.1186/s13023-019-1099-0

**Published:** 2019-05-30

**Authors:** Giovanna Russo, Lucia De Franceschi, Raffaella Colombatti, Paolo Rigano, Silverio Perrotta, Vincenzo Voi, Giovanni Palazzi, Carmelo Fidone, Alessandra Quota, Giovanna Graziadei, Antonello Pietrangelo, Valeria Pinto, Giovan Battista Ruffo, Francesco Sorrentino, Donatella Venturelli, Maddalena Casale, Francesca Ferrara, Laura Sainati, Maria Domenica Cappellini, Antonio Piga, Aurelio Maggio, Gian Luca Forni

**Affiliations:** 1 Oncoematologia Pediatrica, Azienda Policlinico-Vittorio Emanuele, Università di Catania, Via Santa Sofia 78, 95123 Catania, Italy; 2 Dipartimento di Medicina, Sezione Medicina Interna, Università di Verona, Policlinico GB Rossi, AOUI, Verona, Italy; 3 Clinica di Oncoematologia Pediatrica, Dipartimento della Salute della Donna e del Bambino Azienda Ospedaliera, Università di Padova, Padova, Italy; 4 U.O.C Ematologia e Malattie Rare del Sangue e degli Organi Ematopoietici-P.O. Cervello Palermo, Palermo, Italy; 5 Dipartimento della Donna, del Bambino e di Chirurgia Generale e Specialistica, Università̀ degli Studi della Campania “Luigi Vanvitelli”, Napoli, Italy; 6 Dipartimento di Scienze Cliniche e Biologiche, Università di Torino, Ospedale San Luigi Gonzaga, Orbassano, Italy; 7 Dipartimento Integrato Materno Infantile U. O. Complessa di Pediatria Università degli Studi di Modena e Reggio Emilia, Modena, Italy; 8 Unità operativa semplice Studio Emoglobinopatie Simt, Ragusa, Italy; 9 U.O.S talassemia PO Vittorio Emanuele, Gela, Italy; 10 UOC di Medicina Generale, Centro Malattie Rare Fondazione IRCCS Ca’ Granda Ospedale Maggiore Policlinico Pad, Granelli, Milano, Italy; 11 Dipartimento di Scienze Mediche e Chirurgiche Materno-Infantili e dell’Adulto, Università degli Studi di Modena e Reggio Emilia, Modena, Italy; 12 Centro della Microcitemia e delle Anemie Congenite, Ospedale Galliera, Via Volta 6, 16128 Genova, Italy; 13 U.O. Ematologia con Talassemia ARNAS Civico Di Cristina Benfratelli, Palermo, Italy; 14 U.O. Talassemici Centro Anemia Rare e Disturbi del metabolismo del Ferro ASL ROMA 2 Ospedale S Eugenio, Roma, Italy; 15 Struttura Complessa di Immuno-trasfusionale Azienda Ospedaliero, Universitaria di Modena, Modena, Italy; 16 Struttura Complessa di Pediatria-Microcitemie dell’Ospedale San Luigi di Orbassano, Orbassano, TO Italy

**Keywords:** Hemoglobin disorder, Hemoglobinopathy, Hydroxyurea, Migrants, Sickle cell screening, Sickle cell disease, Transfusion, Vaso-occlusion crisis

## Abstract

Sickle cell disease (SCD) is an inherited red blood cell disorder caused by a structural abnormality of hemoglobin called sickle hemoglobin (HbS). Clinical manifestations of SCD are mainly characterized by chronic hemolysis and acute vaso-occlusive crisis, which are responsible for severe acute and chronic organ damage. SCD is widespread in sub-Saharan Africa, in the Middle East, Indian subcontinent, and some Mediterranean regions. With voluntary population migrations, people harboring the HbS gene have spread globally. In 2006, the World Health Organization recognized hemoglobinopathies, including SCD, as a global public health problem and urged national health systems worldwide to design and establish programs for the prevention and management of SCD. Herein we describe the historical experience of the network of hemoglobinopathy centers and their approach to SCD in Italy, a country where hemoglobinopathies have a high prevalence and where SCD, associated with different genotypes including ß-thalassemia, is present in the native population.

## Introduction

The term sickle cell disease (SCD) encompasses a group of inherited red blood cell disorders caused by a structural abnormality of hemoglobin (Hb) called sickle hemoglobin (HbS), which originates from a single nucleotide substitution in the gene encoding ß-globin [1]. HbS is inherited in an autosomal recessive way and SCD can occur due to homozygosity for HbS (HbSS), a condition also known as sickle cell anemia (SCA), or due to compound heterozygosity with ß-thalassemia mutations (HbS/ß^0^-thalassemia and HbS/ß^+^-thalassemia, previously known as microdrepanocytic disease and first described by Silvestroni and Bianco in 1944 [2]), and other ß-globin structural variants such as HbC (HbSC disease) [1]. HbS is functional and soluble when oxygenated, but upon deoxygenation it polymerizes, leading to the generation of misshapen red blood cells known as sickled cells and dense erythrocytes [3]. Sickle red blood cells show: (i) abnormal membrane mechanical stability; (ii) increased membrane oxidation; (iii) activation of pro-dehydrating membrane transport pathways; and (iv) pro-adhesive molecules. The dense, rigid red blood cells are easily trapped within organs with sluggish microcirculation by their interaction with the inflammatory activated vascular endothelial cells and neutrophils. These events generate acute vaso-occlusive events, which leads to ischemic-reperfusion damage of target organs such as lung, kidney or brain [3–6].

Up to now, SCD remains an invalidating chronic disorder with high mortality and morbidity [7, 8]. The most common acute manifestations of SCD include acute hemolytic crisis and vaso-occlusive crisis (VOCs). VOCs are characterized by musculoskeletal pain, which might develop into severe form such as acute chest syndrome, stroke or priapism [1, 3, 9–12]. In addition, with the spleen being one of the target organs of VOC, patients with SCD are also prone to serious bacterial infections due to asplenism [1, 3]. The recurrent pattern of VOCs results in chronic organ damage, which becomes clinically evident in adult patients [13]. SCD was long regarded as a disease of children, with few surviving to adulthood [14]. Today, thanks to advances in infection control, vaccination and screening programs, as well as intensive disease management, more than 95% of children with SCD in developed countries reach adulthood [15]. In adults with SCD, survival is estimated to be over 50 years for patients with HbSS or HbS/ß^0^-thalassemia genotypes, while the survival of patients with HbSC or HbS/ß^+^-thalassemia genotypes is close to that of the general population [15].

Epidemiologic and global burden of disease studies have shown that SCD is widespread in sub-Saharan Africa, in the Middle East, Indian subcontinent and some Mediterranean regions. In the last few decades, due to voluntary population migrations, the HbS gene has spread all over the world. A study published in 2014 estimated that the global number of migrants with HbS increased from approximately 1.6 million in 1960, to 3.6 million in 2000 [16]. In 2006, the World Health Organization (WHO) recognized hemoglobinopathies, including SCD, as a global public health problem and urged national health systems worldwide to design and establish programs for the prevention and management of SCD [17]. The European Union considers SCD a rare disease.

Changes in the demographic profile of SCD have been also reported in Italy [18–20], a country where SCD, in particular HbS/ß-thalassemia, is historically present in the native population. We describe here the experience of new challenges posed to Italian health providers by the increasing prevalence of SCD. To this purpose, we will first discuss the changing epidemiology of SCD in Italy, then we will briefly review the peculiarity of the Italian treatment strategy.

### Epidemiology of sickle cell disease in Italy

The prevalence of SCD throughout Italy is changing and the presence of immigrants in the increasing number of SCD patients in Italian regions with a historically low disease prevalence has been documented by recent studies [19–23]. The highest frequency of the sickle cell allele in Italy was reported in Sicily, with an estimated mean frequency of 2% and peaks as high as 13% [24]. Notably, in Western Sicily SCD appears to have originated from Africa, with chromosomal analysis of the HbSS and HbS/ß-thalassemia genotypes suggesting that the HbSS genotype found in Sicily arrived initially from North African populations [25].

In an Italian survey of 696 cases of SCD, conducted in the late 1990s, 518 cases (74%) were identified as compound heterozygous HbS/ß-thalassemia, 149 cases (21%) as homozygous HbSS, and 21 cases (3%) as compound heterozygous HbS/other Hb structural variant [26]. Of the 673 cases of SCD with a known place of residence, 60% were living in Sicily, 20% in South Italy, 6% in Central Italy, and 13% in North Italy. Hence the survey revealed that, in the 1990s, the majority of SCD patients in Italy resided in Sicily and that they mostly had HbS/ß-thalassemia. A survey update, published in 2003, found that the proportion of SCD patients living in North Italy had increased to 20%, but in Sicily this had decreased to 53% [18]. Furthermore, the proportion of patients with HbSS had increased from 21% in 1998 to 28% in 2003. A comparison of patients of non-Italian versus Italian origin showed that non-Italian patients were mostly homozygous for the HbS allele (72% vs 18%, respectively), were younger (75% <18-years old vs 23%) and lived predominantly in North Italy (61% vs 11%).

Real-life experience with the use of hydroxyurea (HU) in SCD was assessed using data from a retrospective Italian nationwide survey of SCD patients with heterogeneous descent, which registered 1,638 patients. From a total of 652 patients who had received HU during their disease course, 400 patients (64%) were Caucasian in origin and 221 patients (36%) originated from Africa [20]. It is also apparent that the genotype of the Hb allele in Italy is changing over time with an increasing frequency of the homozygous HbSS genotype. Screening programs initiated in the 1970s in Italy have increased public awareness of thalassemia and aided its prevention in target populations as well as enabling screening for other hemoglobinopathies [27–29]. These programs, which aim to prevent hemoglobinopathies, have significantly reduced the frequency of live births with SCD [27–29]. In Sicily, an 85% decrease in the incidence of thalassemia major and SCA (from 1 in 245 live births to 1 in 2,000) has been documented following 30 years of preventative actions, which included legislative action, a public awareness campaign, screening and carrier diagnostics, genetic counselling, and prenatal diagnosis [29]. In addition, a universal screening program for hemoglobinopathies, which includes voluntary pregnancy termination within the 22^nd^ week in case of an affected fetus, is active for couples before and/or after conception according to the Italian law since Italy is considered an area endemic for hemoglobinopathies [30].

The identification of SCD in refugees at their first admission to an emergency department for an acute disease-related event was assessed in a study coordinated by the Italian Society of Thalassemia and Hemoglobinopathies (SITE). In total, 67 patients with SCD (48% children, mostly with the HbSS genotype) were identified from a retrospective analysis of data collected from 2014 to 2017 [31]. The main causes of access to the emergency department were VOC (35.8%), anemia (19.4%), and fever (7.5%); 60% of the identified SCD patients were then followed in reference centers for hemoglobinopathies.

Together, these data suggest that the increased number of patients with SCD in Italy has mostly resulted from migratory patterns of immigrants arriving, in recent years, from countries in which there is a high disease prevalence and that there are approximately 2,000 patients with SCD currently living in Italy.

### Management of patients with sickle cell disease in Italy

The creation of evidence-based guidelines for SCD, as for other uncommon or neglected diseases, has proven challenging due to the complex clinical expression of the disease, and the availability of clinical trials regarding only some screening, management, and monitoring issues of SCD. Notably, an important goal of SCD guidelines is to improve the awareness of SCD and increase the number of health professionals able to provide care for patients with SCD [32].

Effort undertaken over the past 10 years by scientific societies involved in the care of pediatric and adult patients with SCD (the Italian Association of Hematology and Pediatric Oncology [AIEOP] and SITE) has been to develop guidelines for the management of children and adults with SCD, respectively, tailoring international recommendations to the Italian health care system.

In general, currently available guidelines deal with three main areas of SCD management: prevention of infections, stroke, and management of acute and chronic complications; treatment of the various complications (VOC, pain, infections, worsening of anemia, acute chest syndrome, vasculopathy); and specific treatment of severe disease with disease-modifying therapies (transfusion and HU) [32–37].

Preventive measures have a central position in the management of SCD. Prophylactic antibiotics should be initiated as early as 2 months of age in infants and a specific vaccination program should be followed in order to widen the protection against encapsulated bacteria [38, 39].

Primary prevention of stroke is performed by screening children, aged 2 to 16 years, with transcranial Doppler ultrasonography; this allows the identification of children at high risk of stroke, who are placed on transfusions and, eventually, HU for stroke prevention [40].

The use of chronic transfusions (typically given on a monthly basis) to reduce the percentage of HbS in the blood have proven effective in preventing most complications of SCD, including stroke, and in minimizing chronic anemia with significant improvements in the patient-centered outcome of health-related quality of life [1, 35, 41–44]. Preliminary results from the “National Transfusion Treatment Survey in patients with SCD” (NCT03397017), a prospective longitudinal systemic study designed to assess the therapeutic approach in a large Italian cohort of patients with SCD (n=1,579), were reported recently [19]. This national survey, which was coordinated by SITE in collaboration with the Italian Society of Transfusion Medicine and Immunohematology (SIMTI) and AIEOP, showed that 14% of patients are on regular transfusion, while 40% received both transfusion and HU, the transfusional approach being similar in HbSS, HbS/β°-thalassemia and HbS/β^+^-thalassemia patients, who were predominately Caucasian or African; severe VOCs and symptomatic anemia were the main reasons for transfusion. The rate of red blood cell alloimmunization was comparable to the lower rate reported in the literature [45]. Elderly Caucasian SCD patients and their long-term follow-up represent a unique population of SCD, and are extremely informative on aging with SCD.

The introduction of chronic transfusion in children with cerebrovascular disease as well as the aging of adults with SCD has increased the use of different transfusion regimens (e.g.: simple transfusion, erythrocytapheresis, red blood cell exchange), which might lead to iron overload. The availability of iron chelators, including deferoxamine, given parenterally, and the oral agent deferasirox, has definitively improved clinical management of iron-overload in SCD subjects [46–48]. Notably, long-term iron chelation therapy with deferiprone was associated with a similar efficacy and safety profile to that of deferoxamine in patients with SCD and may therefore represent an effective long-term treatment option [49]. However, it is of note that the oral chelator deferiprone is currently off-label.

In Italy, there is also wide experience in the treatment of SCD with erythrocyte exchange as a means of lowering HbS levels, particularly in patients who do not tolerate or are unresponsive to HU. Indeed, manual or automatic red blood cell exchange was shown to be safe and effective in preventing complications of SCD for up to 29 years, with minimal development of iron overload and no increased risk of procedure-related complications in both adult and pediatric SCD patients [50–52]. More recently, an Italian double-center retrospective cross-sectional study showed that early prophylactic erythrocytapheresis (initiated at 10.7 ± 5.2 weeks of gestation) improved maternal and fetal outcomes in SCD women with a history of severe SCD-related organ complications [53]. The generation of SITE recommendations for transfusion strategies in hemoglobinopathies has contributed to standardizing the procedures and sharing the clinical indication to the different transfusional approaches at a national level [54]. The real-life transfusion strategy is the object of a very recent survey [19]. A large observational study coordinated by SITE demonstrated the efficacy of direct-acting antiviral drugs in the eradication of hepatitis C virus (HCV) in an Italian cohort of SCD patients infected with HCV (n=136; 93.5% of these patients achieved a sustained virologic response), which likely occurred due to transfusion therapy prior to the introduction of blood-donor screening in the 1990s [55].

A large body of preclinical and clinical evidence has demonstrated that HU reduces the morbidity and mortality of both adults and children (including infants) with SCD, with a favorable tolerability profile and without significant short-term and long-term safety concerns [56–68]. A series of real-life reports documenting the use of HU in Italy have recently been published [20, 22, 69]. HU was shown to be beneficial as a treatment option in a retrospective, nationwide cohort study of 1,638 patients with SCD of whom 652 patients had received HU during their disease course [20]. Notably, only 39.8% of patients (652 out of 1,638 patients) with SCD who had attended treatment centers across Italy were treated with HU, suggesting its underutilization in clinical practice. The percentage of HbS/β°-thalassemia and HbS/β^+^-thalassemia patients on HU was even lower: 90/624 (14.4%) [19].

A sub-analysis of pediatric data from the retrospective, nationwide cohort study [20] demonstrated the tendency to treat children with lower doses than those recommended; furthermore, although national pediatric guidelines recommend starting HU treatment as early as the first months of life, this cohort of children revealed that HU was never started before 11 months of age [22]. Nonetheless, the results of this survey highlight good adherence to the Italian National Guidelines that included detailed recommendations for the use of HU, which is noteworthy considering patients were mainly first-generation immigrants, who may be socially, culturally, and economically vulnerable [22].

Since VOC are the most common acute manifestations of SCD, the main symptom of which is severe pain, we developed an integrated algorithm to manage acute VOC in the emergency department (ED). In this algorithm we introduced multimodal analgesia as an innovative approach to treat SCD-related pain [35, 70, 71]. Multimodal analgesia allows for a: 1) reduction of side effects since each molecule is used at a lower dosage to obtain the same degree of analgesia of a single molecule at a higher dosage;2) synergistic effect due to blockage of different mechanisms of pain generation; opioid or opioid-like actin modulates central nervous system pain perception whereas non-steroid anti-inflammatory drugs modulate pain of vascular origin and neuroinflammatory-mediated pain (ischemia/reperfusion tissue injury) [70, 71]. This approach is particularly important to prevent addiction to opioids, which is a risk of prescribing these drugs for the treatment of VOC-related pain in SCD.

#### Italian guidelines for the management of children and adults with SCD

AIEOP published comprehensive and detailed guidelines for the management of SCD in children in 2013 [33, 72] and, in 2014 SITE issued recommendations for the treatment of adults with SCD [36]. These recommendations have been conceived as easy-to-consult and practice-oriented guidelines addressing the following topics: prevention of infections and follow-up; treatment of acute events (painful VOC, acute chest syndrome, abdominal pain with biliary dysfunction, infarct or acute cerebrovascular events, priapism, acute anemia and aplastic crisis); management of chronic complications (pulmonary hypertension, bone and joint complications, renal complications, and eye complications); intensive treatment (HU and transfusion plus iron chelation).

In addition, great care has been taken by SITE to develop guidelines devoted to complications, such as bone disease, which are typically observed both in adult and pediatric SCD populations [73].

#### Interactive algorithm for the management of acute events in the emergency department

**Fig. 1:**
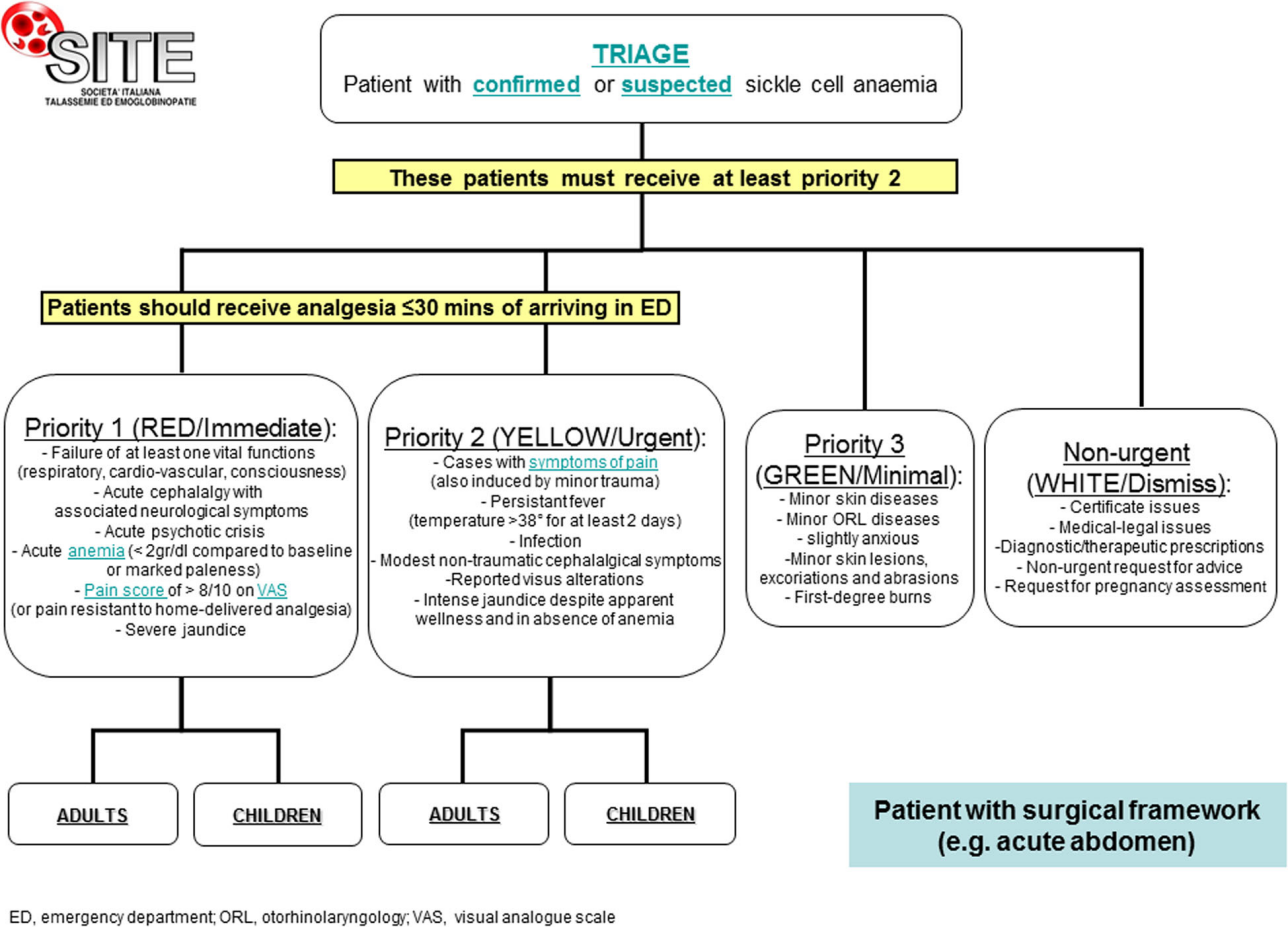
Algorithm for the management of patients with sickle cell disease in the emergency department. Figure reproduced with permission from Forni et al. 2014 [35]

SITE coordinated a panel composed of AIEOP, SIMTI, SIMI (Italian Society of Internal Medicine), and SIMEU (Italian Society of Emergency Medicine) and a representative of the nurses to develop an interactive, easy-to-use algorithm for the clinical management of acute events related to SCD in the ED (Figure 1) [35, 74]. The development of guidelines for the triage and management of SCD patients in the ED responds to the need to improve awareness and knowledge of this hemoglobinopathy, characterized by “time dependent” acute manifestations, among health providers working in this setting where the likelihood of encountering SCD patients will continue to increase [35]. Timely and aggressive interventions, like those for the management of stroke in the general population, are strongly recommended to prevent dramatic evolution of symptoms [35]. Evidence shows that the management of acute VOCs in the emergency department becomes more effective if health providers have received training in SCD [75].

### Measures implemented in Italy to handle the increasing prevalence of SCD

The Italian experience in the management of SCD has grown over the years. In contrast with other countries, this experience has addressed not only HbSS but a number of other genotypes involving ß-thalassemia mutations. Italian centers for hemoglobinopathies have cared for patients with SCD that represent approximately 10% of the global population with hemoglobinopathies. The background knowledge accumulated by these centers, and their distribution throughout Italy, highlights Italy as an example of a successful network of expert physicians on hemoglobinopathies to optimize patient clinical management. This network has been built since the 1960s together with national programs of prevention and screening. Due to the in- and out-migration fluxes towards large urban centers, the distribution of patients with SCD in regions where historically there was a low prevalence of hemoglobinopathies makes it necessary to widen the national network to these regions and to expand the training of specialists and of informed health providers about this multifaceted disease. The need to reinforce the national network of hemoglobinopathy has been addressed by AIEOP and SITE through the creation, improvement and revision of guidelines tailored to Italy’s resources and healthcare system.

In 2017 the Italian government approved a law supporting the institution of a National Thalassemias and Hemoglobinopathies Network [76]. The main objective of this network is to further potentiate the activity of existing clinical centers devoted to hemoglobinopathies and to improve access of patients to highly specialized and comprehensive treatment. Ideally, this should impact positively on the management of SCD in Italy.

It is generally recognized that registries are important tools for detecting demographic patterns, allocating resources, monitoring patient outcomes, and guiding decisions [23, 77, 78]. The creation of an Italian national registry of hemoglobinopathies (Registro nazionale della talassemia e delle emoglobinopatie [National Registry of Thalassemia and Hemoglobinopathies]) was approved in 2017 [79] due to a strong joint action of SITE and national patients associations, and the first national epidemiologic data should be available soon.

AIEOP and SITE have also joined forces to promote programs of newborn screenings [38]. This preventive measure may be redundant in the presence of effective antenatal and/or prenatal screening programs, considering that specific examination for the diagnosis of hemoglobinopathies are freely available from the National Health Service for all child-bearing age individuals and gynecologists screen all pregnant women who are unaware of their status; however, newborn screening becomes crucial for individuals from high-risk ethnic groups who may be unaware of their carrier status as they were never screened for SCD in their country of origin or if there are situations of segregation that cause pregnancy to be outside of the normal care channels. To date, published evidence concerning newborn screening for SCD in Italy is limited to a few regional pilot projects [30, 80–83]. A scientific discussion has been opened by a recent Pan-European consensus conference on newborn screening for SCD, which involved a panel of >50 SCD experts from 13 European countries, and has recently been published [84]. Finally, recent evidence from a retrospective study highlights the importance of SCD screening of refugees, from countries with a high frequency of HbS, on their arrival in Italy [31].

### Research activities carried out over the last 10 years in Italy

Beside the organization of care for patients with SCD, efforts have been made to develop research programs and involve researchers in SCD both in preclinical and clinical studies. This effort was directed on the following topics: genetic aspects of the disease, and its possible reciprocal influence with other genes [85]; mechanisms related to the molecular pathophysiology of the disease [5, 86–94]; prenatal diagnosis, exploring either methods or clinical impact [29, 95]; iron monitoring [49]; chronic organ damage monitoring, either liver [96, 97], kidney [98], heart [99] or brain [100–103]; cognitive function [104, 105].

## Conclusions and perspectives

Overall, the Italian historical experience regarding the global care of hemoglobinopathies is an example of adherence to the recommendations of the WHO to implement comprehensive national programs for the prevention and management of SCD. Alongside this is the institution of the National Thalassemias and Hemoglobinopathies Network [76], which is designed to further potentiate the activity of existing clinical centers devoted to hemoglobinopathies, to improve access of patients to highly specialized and comprehensive treatment in Italy, and to improve the coordination of initiatives and access to new therapeutical approaches. In support of this activity, a number of easy-to-use, practice-oriented and detailed recommendations have been developed by National Scientific Societies. These will no doubt increase the awareness and understanding of SCD among Italian clinicians and further optimize the management of this complex and severe condition. These are ambitious, but feasible, goals and further effort will be required from all professionals involved in the management of SCD. It is possible that the availability of novel drug therapies (crizanlizumab, voxelotor, l-glutamine), bone marrow transplant alternatives from the typical familiar donor (haploidentical, matched unrelated donor), and gene therapy may change the clinical outcome of SCD. As such, a sound national network can facilitate the access of patients to the most appropriate treatment.

## Data Availability

Not applicable

## Abbreviations

AIEOP: Italian Association of Hematology and Pediatric Oncology
Hb: Hemoglobin
HbS: Sickle hemoglobin
HU: Hydroxyurea
SCA: Sickle cell anemia
SCD: Sickle cell disease
SIMEU: Italian Society of Emergency Medicine
SIMI: Italian Society of Internal Medicine
SIMTI: Society Italian Transfusion Medicine and Immunohematology
SITE: Italian Society of Thalassemia and Hemoglobinopathies
VOC: Vaso-occlusive crisis
WHO: World Health Organization
